# Using systematic reviews to inform NIHR HTA trial planning and design: a retrospective cohort

**DOI:** 10.1186/s12874-015-0102-2

**Published:** 2015-12-29

**Authors:** Sheetal Bhurke, Andrew Cook, Anna Tallant, Amanda Young, Elaine Williams, James Raftery

**Affiliations:** Wessex Institute, University of Southampton Alpha House, University of Southampton Science Park, Southampton, SO16 7NS UK; National Institute for Health Research (NIHR), Evaluation, Trials and Studies Coordinating Centre (NETSCC), University of Southampton, Southampton, SO16 7NS UK; University of Southampton and University Hospital Southampton NHS Foundation Trusts, Southampton, UK

**Keywords:** Systematic reviews, Health technology assessment, Randomised controlled trials, Design, Primary outcome

## Abstract

**Background:**

Chalmers and Glasziou’s paper published in 2014 recommends research funding bodies should mandate that proposals for additional primary research are built on systematic reviews of existing evidence showing what is already known. Jones et al. identified 11 (23 %) of 48 trials funded during 2006–8 by the National Institute for Health Research Health Technology Assessment (NIHR HTA) Programme did not reference a systematic review. This study did not explore the reasons for trials not referencing a systematic review or consider trials using any other evidence in the absence of a systematic review. Referencing a systematic review may not be possible in certain circumstances, for instance if the systematic review does not address the question being proposed in the trial. The current study extended Jones’ study by exploring the reasons for why trials did not reference a systematic review and included a more recent cohort of trials funded in 2013 to determine if there were any changes in the referencing or use of systematic reviews.

**Methods:**

Two cohorts of NIHR HTA randomised controlled trials were included. Cohort I included the same trials as Jones et al. (with the exception of one trial which was discontinued). Cohort II included NIHR HTA trials funded in 2013. Data extraction was undertaken independently by two reviewers using full applications and trial protocols. Descriptive statistics was used and no formal statistical analyses were conducted.

**Results:**

Five (11 %) trials of the 47 funded during 2006–2008 did not reference a systematic review. These 5 trials had warranted reasons for not referencing systematic reviews. All trials from Cohort II referenced a systematic review. A quarter of all those trials with a preceding systematic review used a different primary outcome than those stated in the reviews.

**Conclusions:**

The NIHR requires that proposals for new primary research are justified by existing evidence and the findings of this study confirm the adherence to this requirement with a high rate of applications using systematic reviews.

**Electronic supplementary material:**

The online version of this article (doi:10.1186/s12874-015-0102-2) contains supplementary material, which is available to authorized users.

## Background

The use of systematic reviews to identify gaps in health related research is well documented and should be conducted, with or without a meta analyses, to assess existing evidence and the need for further primary research [1]. Chalmers and Glasziou’s paper on how to increase value and reduce waste when research priorities are set identified from surveys of previous reports of clinical trials that existing research was being ignored. The study identified from an analysis of 50 reports including more than 1500 cumulative meta-analyses of clinical intervention studies that if researchers had systematically assessed what was already known some beneficial and harmful effects of treatments could have been identified earlier. In these cases systematic reviews would have reduced waste resulting from unjustified research. The authors provided a number of recommendations for research funders, most notably that proposals for additional primary research are justified by systematic reviews showing what is already known [2]. If new research is needed it should be justifiable both scientifically and ethically and needs to be planned in the context of an assessment of relevant previous research, ideally through a systematic review [3, 4]. As such, efforts should be taken to understand and improve the production of research in its entirety.

Systematic reviews provide a synthesis of evidence for practitioners, for clinical practice guideline developers, and for those designing and justifying primary research. It is therefore important to have an up-to-date review. However, previous work by Clarke and Hopewell has shown that very few investigators state that they had used up-to-date systematic reviews when designing their new clinical trials [5]. Further research has shown the extent of the lack of use of systematic reviews in planning the targeted sample size calculations [6] as well as the design of the interventions [7].

In 2013, Jones et al. investigated the use of systematic reviews in the planning, design and conduct of randomised trials funded by the NIHR HTA Programme during 2006, 2007 and 2008 [8]. Although the study pre-dates the Chalmers and Glasziou 2009 Lancet publication, the findings provide a useful insight to the clinical relevance of the research question and appropriateness of the trial design. The study concluded that 37 HTA applications (77 %) out of 48 referenced a systematic review and 20 of these 37 applications (54 %) reported the use of systematic reviews in the trial design [8]. Eleven (23 %) out of the 48 applications made no reference to systematic reviews and of these 7 (15 %) stated that there had been no previous trials and one explicitly stated the absence of a systematic review. The reasons for why these trials did not reference a systematic review was however was absent from Jones et al. study. The paper did not consider that in the absence of a systematic review there might have been other evidence confirming the rationale for the trial. Since only half of trials assessed in Jones et al. used a systematic review to inform the trial design, the current study chose to investigate this issue further. The purpose of our study was to firstly replicate the Jones et al. study and secondly explore, a. the reasons why some trials did not reference a systematic review, b. provide a classification system for inclusion and c. extend and include a more recent cohort of NIHR HTA-funded trials (2013) post Chalmers and Glasziou 2009 Lancet publication to determine any improvements over time.

## Methods

**Fig. 1:**
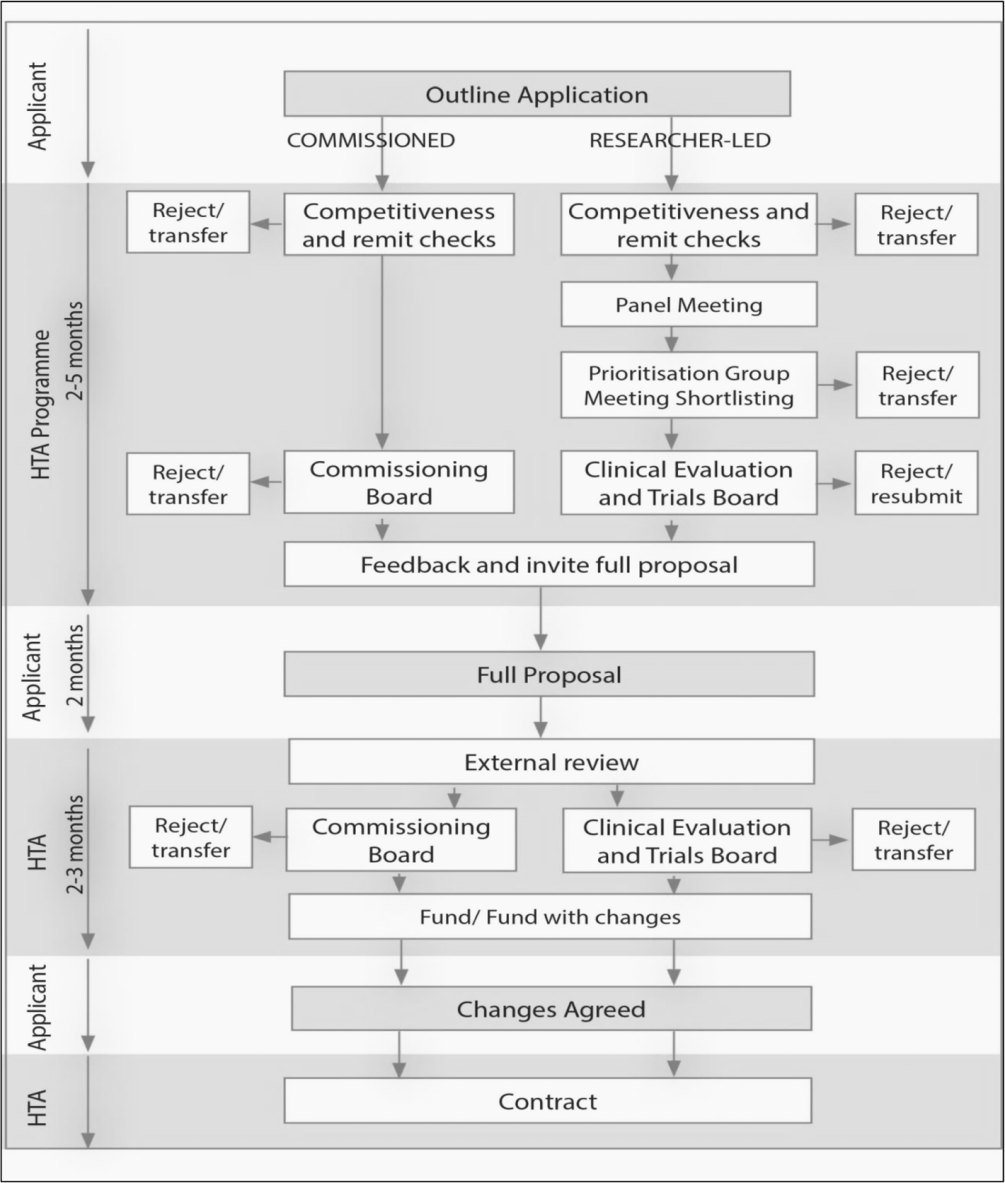
Funding process for the NIHR HTA programme

Two cohorts of randomised controlled trials funded by the NIHR HTA Programme were used. The first cohort included the same NIHR HTA trials funded in 2006, 2007 and 2008 as that of Jones et al. [8] (with the exception of one trial which was discontinued in 2009 due to problems with recruitment). We extended Jones et al’s analysis by exploring the reasons why some trials did not reference a systematic review. The second cohort included NIHR HTA trials funded in 2013 to explore whether changes made by the HTA programme towards the content of the applications, has had an impact on how systematic reviews are used to inform trial design. The funding process for NIHR HTA trials comprises several stages (Fig. 1). Researchers can apply for funding for their own research idea through the researcher-led workstream or through the commissioned workstream whereby researchers apply for funding in response to an advertised brief in areas identified as important to the NHS by the HTA prioritisation panel.

The list of relevant HTA trials funded during the periods 2006 to 2008 and 2013 were identified by the HTA funding team senior programme manager. We had full access to the relevant documentation, including outline, full application, protocol, board minutes and comments from experts. The protocols for all included studies were publicly available and the remaining documents we used can be obtained by bona-fide researchers on application. From both the cohorts, studies that have been published were freely available but for the studies that were still in the editorial process, we accessed the final draft reports. The full application and the most current trial protocol were predominately used as the main source of information, unless otherwise stated.

An Access database was developed and designed for the purposes of the project (AY). This was piloted and amended where necessary (SB and AT). The data extraction form was developed based on Jones et al. [8] to include data on whether systematic reviews were referenced and used in the design of trials for justification of treatment comparison, choice of frequency or dose, selection of an outcome, withdrawals or adverse events etc (refer to Jones et al. for a full listing). We developed the criteria for use of systematic reviews to inform the trial design (see Additional file 1). Data extraction was independently undertaken by two reviewers (SB and AT) and any disagreements were resolved by discussion with a third reviewer (AC). In the absence of reference to a systematic review we explored whether additional evidence was used to support the rationale for conducting primary research either from the application or from the commissioning brief if the study was funded through the commissioning workstream.

We defined a systematic review as previously existing if we found, relating to the trial research question, one or more of the following:

- a Cochrane systematic review
- other reviews if systematic review was mentioned in the title and methods stated a systematic search was conducted
- a National Institute for Health and Care Excellence (NICE) Technology Appraisal Guidance (TA) which include the Technology Assessment Report (TAR) based on the review of clinical and economic evidence

All data were descriptive and no formal statistical analyses were conducted. Data were stored and reported using Microsoft Excel 2010.

## Results

**Fig. 2:**
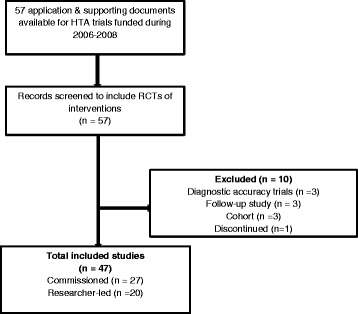
Cohort I – HTA trials funded during 2006–2008

We included 47 trials funded by the NIHR HTA Programme in 2006, 2007 and 2008 in the first cohort. Of these 27 were from the commissioned and 20 from the researcher-led workstream (see Fig. 2).

**Fig. 3:**
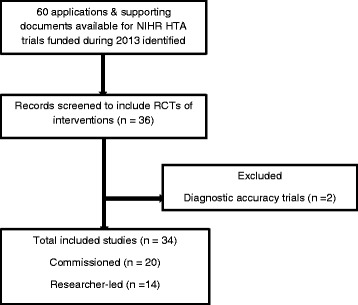
Cohort II – HTA trials funded during 2013

The second cohort included 34 trials funded in 2013, of which 20 were from the commissioned and 14 from the researcher led workstream (see Fig. 3).

Five trials from Cohort I did not reference or use a systematic review to inform their design (Table 1). All trials from Cohort II referenced a systematic review. Additional file 2 lists the five trials from Cohort I and the reasons why they did not reference a systematic review.

**Table 1:** Trials with no reference or use of systematic reviews

Total number of trials	No. of trials not referencing or using a systematic review to inform their design	Justified reasons for not referencing and using a systematic review to inform the trial design (n)
47 – Cohort I	5	5
34 – Cohort II	0	0

### 2006-2008 - Cohort I

This cohort comprised of the same 47 NIHR HTA-funded trials that Jones et al. included in their study with the exception of one trial which has been discontinued since Jones et al. published their study. Of these 42 (89 %) trials used a systematic review to inform their design and of these 30 trials (71 %) referenced more than one systematic review, with a total of 109 systematic reviews being referenced. Twenty seven (25 %) of these 109 systematic reviews had been published in the Cochrane Database of Systematic Review (CDSR) and the remaining 82 (75 %) in other peer reviewed journals.

#### Trials reporting no systematic review

The five trials that did not use a systematic review to inform their design were all from the researcher-led workstream. For two of these trials, NICE TAs (always informed by a systematic review) relating to that specific drug or intervention had been published before the funding of the HTA trial. The assumption is that the HTA funding board would have discussed the evidence relating to the specific drug before the HTA trial was funded. Also, the HTA programme always runs updated searches before the proposal is presented at the funding board to ensure no recent research has been missed or emergence of new evidence may influence the funding decision. For the HTA trial PERSEPHONE (06/303/98) shortlisted for the HTA funding board in 2006, the NICE TA No. 34 (2002) reported the evidence for the effectiveness of trastuzumab monotherapy to be limited and that there was insufficient evidence on the length of follow-up [9]. The evidence for NICE TA No. 34 was prepared by the NHS Centre for Reviews and Dissemination. In addition, a HTA systematic review (Lewis 2002), investigating the clinical effectiveness of trastuzumab for breast cancer also suggested further research to evaluate the optimum duration of the therapy [10]. For the HTA trial TRAPEZE (06/303/205) shortlisted for the HTA funding board in 2006, the NICE TA No. 101 published in June 2006 identified a need for research to assess the quality of life associated with different treatments for hormone-refractory metastatic prostate cancer using generic quality of life instruments that are suitable for the purposes of cost-effectiveness analyses [11]. The report also identified a need for research on the effects of docetaxel over a longer follow-up period, and in patient group that is more representative of a wider patient population in terms of age, performance status and comorbidity. The evidence to support the NICE TA No. 101 was prepared by the Systematic Reviews Centre for Reviews and Dissemination [12].

In the HTA trial AESOPS (06/304/142) the applicants identified five systematic reviews but these did not cover older patients. The five systematic reviews identified focussed specifically on the effectiveness of brief interventions in primary care populations.

A systematic review may have been underway when the HTA trial (06/403/90) of protease inhibitor monotherapy versus continuing combination antiretroviral therapy for HIV-1 infected patients was funded. This study is currently being edited for publication by the NIHR Journals Library and has cited a systematic review published in 2011 [13].

The HTA CHAMP trial (07/01/26) was the first large scale trial of cognitive behaviour therapy for health anxiety in secondary care. The application states that there were increasing studies on the clinical benefits of this treatment in primary care but the added value of this treatment in secondary care in terms of cost-effectiveness was uncertain with no evidence on the importance of 2 year follow-up. This study was the first-in-class (Additional file 3).

#### Trials reporting the use of a systematic review

Of the trials (*n* = 42) that reported using a systematic review in the design or planning stages, 30 trials referenced more than one systematic review. Therefore more than one reason has been included in the assessment (Table 2). We identified the use of systematic reviews in 40 out of 42 trials from the full application and trial protocol. The remaining two trials, (07/36/01 – A randomised controlled trial (RCT) of alternative treatments to Inhibit VEGF in patients with Age-related choroidal Neovascularisation (IVAN) and 07/39/01 - Amnioinfusion in preterm premature rupture of membranes (AMIPROM): a randomised controlled trial of amnioinfusion versus expectant management in very early preterm premature rupture of membranes - a pilot study), were funded through the commissioned workstream, which means the HTA programme would have completed a robust search to identify the evidence gap and determined the value of any systematic review towards the justification of treatment. Taking each of these trials in turn, the applicants of the HTA trial 07/36/01 stated that they searched the literature but found no previous trials or reviews. We independently reviewed the background to this trial and found that the drug in question had never been subject to a randomised trial for the specified condition. This trial was the first-in-class (Additional file 3).

**Table 2:** The use of systematic reviews in trial design

Reasons	Cohort I No. of applications (%)	Cohort II No. of applications (%)
	(*n* = 42)	(*n* = 34)
Adverse events	7 (16.6)	1 (2.9)
Choice of frequency/dose	2 (4.7)	1 (2.9)
Duration of follow-up	1 (2.3)	2 (5.8)
Estimating the control group event rate	2 (4.7)	0 (0)
Estimating the difference to detect or margin of equivalence	2 (4.7)	1 (2.9)
Inform standard deviation	0 (0)	3 (8.8)
Intensity of interventions	1 (2.3)	1 (2.9)
Justification of prevalence	3 (7.1)	0 (0)
Justification of treatment comparison	38 (90.4)	32 (94.1)
Recruitment and consent	4 (9.5)	1 (2.9)
Route of intervention	0 (0)	1 (2.9)
Selection of definition or outcome	5 (11.9)	5 (16.1)
Withdrawal rate	1 (2.3)	0 (0)

For HTA trial 07/39/01, the applicants state the various observational comparative studies they identified and also discuss about a very small randomised trial of 19 women. The applicants state that further research was recommended by NICE. Clinical guidance issued by NICE are based upon a comprehensive literature review including a systematic review and various clinical and stakeholder consultations. Research recommendations, which form part of the clinical guidance documents, are made based on a recognised evidence gap in that area.

#### Primary outcomes

Fourteen (33 %) trials out of 42 that referenced a systematic review used a different primary outcome to that used in the review. The reasons for using a different primary outcome were that the outcome was not considered clinically important, a specific primary outcome was requested by the HTA commissioning board or because there were heterogeneous primary outcomes included in the systematic review (Table 3).

**Table 3:** Reasons for using different primary outcome

Reasons	Cohort I No. of applications (%)	Cohort II No. of applications (%)
	(*n* = 14)	(*n* = 8)
Change requested by HTA commissioning board	2 (14.2)	0 (0)
Feasibility/Pilot Study	2 (25)	0 (0)
Heterogeneous outcomes in the review	1 (7.1)	0 (0)
Primary outcome not believed to be clinically important	11 (78.5)	2 (25)
Unclear	0 (0)	4 (50)

In addition to these 14 trials, two trials used a different primary outcome due to the multiplicity of reviews with varying primary outcomes. Therefore it was unclear which review informed the primary outcome.

### 2013 – Cohort II

This cohort included 34 NIHR HTA trials funded during 2013. All trials referenced and used a systematic review to inform the trial design. Of these 20 (59 %) referenced more than one systematic review, with 80 systematic reviews referenced in total. Sixteen (20 %) of these 80 systematic reviews had been published in the CDSR and the remaining 64 (80 %) published in other peer reviewed journals.

#### Trials reporting the use of a systematic review

Of all the 34 trials reporting the use of a systematic review in the design or planning of a trial, 20 referenced more than one systematic review. Therefore more than one reason has been included and Table 2 summarises these reasons. We identified the use of systematic reviews in 32 out of 34 trials from the full application and trial protocol. The first remaining trial (11/148/01) was funded through the HTA commissioned workstream which would have included a robust search to identify the evidence gap, as mentioned previously. The other HTA trial (12/167/135) application stated how NICE recommended the need for the research. The trial was the initial attempt to address the research question using an experimental method, designed to include a systematic review as part of the methodology (12/167/135: Randomised controlled trial to examine the efficacy of e-cigarettes compared with nicotine replacement therapy).

#### Primary outcomes

Eight trials (24 %) out of the 34 used a different primary outcome. For four trials it was unclear why a different primary outcome had been chosen to that used in the referenced systematic review. Two trials established that the primary outcome used in the systematic review was not clinically important and the remaining two trials were feasibility or pilot studies (Table 3).

In addition to the above eight trials, 14 (41 %) trials had a different primary outcome as each of these trials referenced more than one review. It was unclear which review was used to select the primary outcome.

We identified two studies from Cohort I and one study from Cohort II which were first-in-class trials funded by the NIHR HTA Programme. The justifications for these trials being the first-in-class are listed in Additional file 3.

## Discussion

This study replicated Jones et al. [8] study and further explored the reasons why some trials did not reference a systematic review. Our study shows that systematic reviews were not referenced in five (11 %) of the 47 trials funded in the period 2006–2008. All five trials had plausible reasons for not referencing a systematic review. In Cohort II all HTA trials funded during 2013 referenced a systematic review. There appears to be two reasons for not referencing a systematic review in Cohort I; NICE TA were published prior to the trial being funded and the HTA funding board being aware of the body of evidence relating to that specific drug/intervention or systematic review(s) had been identified but did not address the proposed research question.

There is limited evidence about whether systematic reviews are referenced or used in the design and planning of randomised trials. One of the strengths of this study is that it not only explored the reasons for non-reference of a systematic review but also provided a broader overview of the evidence relating to the specific intervention in the absence of reference to a systematic review. Systematic reviews are only used as a proxy measure to understand if the existing evidence has been referred to. Assumptions cannot be made about an application based only on its referencing of a systematic review.

Additionally, a strength of our study was the development of definitions for what constituted as a systematic review and for different reasons for using a systematic review in the design and planning of a trial. Building on Jones’ previous work the current study was able to pilot a classification system which was piloted by one reviewer (SB) and quality assured by another reviewer (AY). Applying the classification to two cohorts has shown that in some instances it is not possible or indeed plausible to reference a systematic review. However, owing to the interpretive nature of the classification system, this could also be regarded as a limitation of this current study.

Previous research shows that little is known about how existing evidence is used to inform the design and reporting of randomised clinical trials. Goudie and colleagues [14] investigated whether authors of trials considered previous trials in their design and reported that previous trial results were consulted in the design of just 37 % of current trials. Cooper et al. found that under half (46 %) of responding authors of new research were aware of relevant reviews when they designed their new studies [15]. Compared to previous research our findings demonstrate that funding applications to the NIHR HTA Programme have a higher percentage of trials referencing systematic reviews.

The HTA programme has two work streams (commissioned and researcher-led) which the current study has identified as an indicator to how different these streams are when assessing the use and application of systematic reviews during the application process. By extending Jones et al. paper to include a clear definition of ‘systematic review’ to determine which reviews should be included may have inadvertently resulted in differences between our study and theirs However, by predefining a description for each area of use these definitions can now be piloted for similar research. The transparency of using predefined definitions demonstrates the importance of proposing the right research question and using appropriate methods.

Using systematic reviews to inform trial design is important and it is recommended that research funders make it a funding requirement to justify the need for new research with existing evidence. The NIHR is now committed to Adding Value in Research [16] to maximize the potential impact of research it funds on behalf of patients and the public. Chalmers and Glasziou in their paper also recommended that research funders and regulators demand that proposals for additional primary research are justified by systematic reviews evaluating what is already known [2]. The NIHR has now included this in application guidelines for their research programmes to ensure that the research they fund answers the right questions and has maximum impact.

## Future research

The current study has shown that NIHR HTA trials use systematic reviews where and when possible. There is a need for better use of systematic reviews in the planning and design of trials, with funders explicitly requesting information about what already exists and how their trial design is based on existing evidence. To maximise the benefit of trial results we now need to ensure that the primary outcome is fit for purpose.

## Conclusions

Systematic reviews are referenced in NIHR HTA trials wherever it is feasible to include a systematic review. Ninety four percent of trials used one or more systematic review(s) in their design and planning. The NIHR HTA Programme requires evidence from systematic reviews before funding primary research and our study shows that this is being implemented by applicants when they apply for primary research funding. Systematic review authors could maximise the impact of trial design by reporting primary outcomes that are relevant to the conduct of a trial.

## Additional files

Additional file 1:
**Criteria for use of systematic reviews to inform trial design.** (PDF 58 kb) Additional file 2:
**Reasons for not referencing and using a systematic review.** (PDF 201 kb) Additional file 3:
**Justification for first-in-class trials.** (PDF 19 kb)

## Abbreviations

CDSR: Cochrane Database of Systematic Review
HTA: Health Technology Assessment
NICE: National Institute for Health and Care Excellence
NIHR: National Institute for Health Research
TA: Technology Appraisal Guidance
TAR: Technology Assessment Report
