# Supplementary material for: Using systematic reviews to inform NIHR HTA trial planning and design: a retrospective cohort
Source: BMC Med Res Methodol. 2015 Dec 29;15:108. doi: 10.1186/s12874-015-0102-2 (PMC4696153; doi:10.1186/s12874-015-0102-2)
Supplement: Additional file 1: — Criteria for use of systematic reviews to inform trial design. (PDF 58 kb) [file 12874_2015_102_MOESM1_ESM.pdf]

Additional file 1 - Criteria for use of systematic reviews to inform trial design

Justification of treatment comparison	If the review identified gap in the evidence or few smaller studies identified need for a trial.
Selection of definition or outcome	If the outcome or a measure for an outcome specified in the review was used in the trial and was justified for doing so.
Recruitment and consent	If the review justified the case for continuing recruitment or provided information on recruitment rates, difficulties etc. which has been used to further inform recruitment in the trial.
Adverse events	If the review provided information on adverse events and this has been used by the trial
Justification of prevalence	If the review justified the prevalence of the disease
Choice of frequency/dose	If the review provided information on choice of frequency/dose of the intervention and that was used by the trial to determine the right frequency/dose
Estimating the difference to detect or margin of equivalence	If the review provided information on effect size and this information has been used by the trial to estimate the effect size
Estimating the control group event rate	If the review provided information on control event rate and the trial used this information

CONFIDENTIAL

	to make an estimate of the control group event rate.
Intensity of interventions	If the review identified that the intensity of intervention was not adequate and the trial used the information to determine the intensity of interventions
Duration of follow-up	If the review provided information on duration of follow-up (short term or long term) and if that information was used in the trial design to determine the required duration of follow-up
Withdrawal rate	If the review provided information on the withdrawal or drop-out rate and if this was used to inform the trial

2

CONFIDENTIAL
