# Supplementary material for: Using systematic reviews to inform NIHR HTA trial planning and design: a retrospective cohort
Source: BMC Med Res Methodol. 2015 Dec 29;15:108. doi: 10.1186/s12874-015-0102-2 (PMC4696153; doi:10.1186/s12874-015-0102-2)
Supplement: Additional file 2: — Reasons for not referencing and using a systematic review. (PDF 201 kb) [file 12874_2015_102_MOESM2_ESM.pdf]

Additional file 2 - Reasons for not referencing and using a systematic review

Project Reference	Work stream – Commissioned (C) or Researcher-led (RL)	Reason for not including a systematic review	Quote from the application
**Cohort I – Trials funded during 2006-200**			
06/303/98	RL	Searched, identified several trials but not relevant<br><br>We identified a related NICE TA which would have been considered at the HTA funding board	"However, the use of 12 months trastuzumab in the majority of studies is not based on evidence. It is reasonable to consider that since the beneficial effect of adjuvant trastuzumab is detected early in follow-up (median 1 year), that the majority of the adjuvant benefits results from the first 6 months of therapy. This hypothesis is supported by evidence from the FinHer study which, with only 9 weeks trastuzumab demonstrates a similar-sized benefit to 12 months treatment, when given

			concurrently with chemotherapy. The Persephone trial will compare 6 months treatment with 12 months, in terms of safety and efficacy.”
06/303/205	RL	Searched and identified several on-going trials but all using new agents to treat HRPC<br><br>We identified a related NICE TA which would have been considered at the HTA funding board	“There are a large number of trials of new agents currently underway in metastatic HRPC and `it is likely that additional effective treatments will become available in the coming years, though it is unlikely that they will supplant the current options.”
06/304/142	RL	Searched, identified five systematic reviews but these did not cover older patients	"There are five systematic reviews focusing specifically upon the effectiveness of brief interventions in primary care populations all conclude that brief interventions in primary care populations are effective in reducing

			alcohol consumption. But many of the studies included in these reviews exclude older patients. There are no systematic reviews or subgroup analyses specifically focussing on older patient groups"
06/403/90	RL	Searched, identified several trials but not relevant<br><br>A systematic review may have been underway when this study was funded. This study is currently in editorial with the HTA Journal and has cited a systematic review published in 2011.	"Although the trials of PI monotherapy described above are encouraging, this research is driven by the pharmaceutical industry with a typical short-term focus. A definitive trial focused on long-term outcomes is lacking. This trial is different in a number of respects from the previously completed or ongoing pharma-sponsored studies."
07/01/26	RL	Searched, identified no previous trials in secondary care	"No randomized controlled trials had been carried out in the population attending

			secondary medical clinics where health anxiety is likely to be more common and has a greater impact on services, until we carried out our pilot trial described below.”
