# Supplementary material for: Using systematic reviews to inform NIHR HTA trial planning and design: a retrospective cohort
Source: BMC Med Res Methodol. 2015 Dec 29;15:108. doi: 10.1186/s12874-015-0102-2 (PMC4696153; doi:10.1186/s12874-015-0102-2)
Supplement: Additional file 3: — Justification for first-in-class trials. (PDF 19 kb) [file 12874_2015_102_MOESM3_ESM.pdf]

Additional file 3 – Justification for first-in-class trials

Cohort I – 2006-2008

HTA Project Reference – 07/01/26

Title - Cognitive-behavioural therapy for Health Anxiety in Medical Patients (CHAMP)

Dates – October 2008 – November 2016

Status – Active

<u>Excerpt from protocol (http://www.nets.nihr.ac.uk/projects/hta/070126)</u>

"*No randomized controlled trials had been carried out in the population attending secondary medical clinics where health anxiety is likely to be more common and has a greater impact on services, until we carried out our pilot trial described below*."

<u>Excerpt from Lancet (Volume 383, Issue 9913, Pages 219-225, 2014)</u>

"In a pilot study carried out by our group these results generalised to secondary medical care settings (in which costs of care are high) using less expert therapists trained for the purpose. We subsequently set up the cognitive behaviour therapy for health anxiety in medical patients (CHAMP) trial to examine both effectiveness and cost-effectiveness of a modified cognitive behavioural treatment for health anxiety (CBT-HA) with assessment of outcomes over a 2 year period."

"The CHAMP trial was the first large scale trial of an adapted form of cognitive behaviour therapy for health anxiety (CBT-HA) which can be taught easily to naive therapists such as general nurses."

Cohort II – 2013

HTA Project Reference – 12/167/135

Title - A randomised controlled trial to examine the efficacy of e-cigarettes compared with nicotine replacement therapy, when used within the UK stop smoking service

Dates – October 2014 – March 2018

Status – Active

Excerpt from National Institute for Clinical Excellence (NICE)

“NICE have called for more data regarding the effects of EC on smoking behaviour. To date, no study has examined the efficacy of EC as a cessation aid compared to the current state-of-the-art treatment. The proposed study will produce data that will guide policy and practice in the UK and internationally. More recently the National Institute for Health and Clinical Excellence (NICE), in their guidance on Tobacco Harm Reduction, highlighted the critical need for outcome and safety research on novel nicotine-containing products, in particular EC.”
